# Maternal deaths and severe maternal morbidity in the Nordic countries—Time to build collaborative routine surveillance and shared learning to improve healthcare

**DOI:** 10.1111/aogs.70376

**Published:** 2026-10-16

**Authors:** Hilde Marie Engjom, Outi Äyräs, Anna Aabakke, Eva Jonasdottir, Teresia Svanvik, Lill Nyfløt

**Affiliations:** ^1^ Department for Health Promotion, Division of Public Health and Prevention The Norwegian Institute of Public Health Bergen Norway; ^2^ Department of Clinical Services and Organization Development Stavanger University Hospital Stavanger Norway; ^3^ Department of Obstetrics and Gynecology Helsinki University Hospital and University of Helsinki Helsinki Finland; ^4^ Department of Obstetrics and Gynecology Copenhagen University Hospital – Holbæk Holbæk Denmark; ^5^ Institute of Clinical Medicine University of Copenhagen Copenhagen Denmark; ^6^ Women's Health Department Landspitali ‐ University Hospital Iceland; ^7^ Institute of Clinical Sciences, Department of Obstetrics and Gynaecology University of Gothenburg Gothenburg Sweden; ^8^ National Research Centre for Women's Health Oslo University Hospital HF Oslo Norway; ^9^ Department of Obstetrics and Gynecology Oslo University Hospital HF Oslo Norway

**Keywords:** learning, maternal mortality, Nordic, severe maternal morbidity, surveillance

The Nordic Maternal Mortality Collaboration and the Nordic Obstetric Surveillance Study (NOSS) collaboration have studied causes of maternal deaths and severe morbidity in the Nordic countries. The groups comprise clinicians and epidemiologists in the five Nordic countries: Denmark, Finland, Iceland, Norway, and Sweden and aim to describe causes of maternal deaths and severe morbidity and facilitate joint learning to improve clinical obstetric care in the Nordic countries.

The Nordic collaborations participate jointly in the International Network of Obstetric Survey Systems (INOSS) with other countries in Europe, North America, Africa, and Asia.^1^

Several European countries have already established routine systems for enhanced surveillance and learning from severe maternal and perinatal outcomes, using the MBRRACE‐UK as the gold standard. Enhanced surveillance entails a combination of several sources to capture all severe adverse events; this is particularly important for maternal deaths and severe, potentially life‐threatening maternal morbidity, where single data sources lead to underreporting.^2^ The benefits have been illustrated by the reduced incidence of maternal deaths and severe morbidity through contributions to national health plans, clinical guidelines and targeted training for health professionals and involvement of community stakeholders.

In the Nordic countries, surveillance and confidential enquiries into maternal deaths and severe morbidity have been organized as individual research projects. The incidence of maternal deaths, severe adverse maternal outcomes and perinatal deaths is comparable across countries.^3^, ^4^ The Nordic maternal mortality groups conduct enhanced surveillance by combining the national Medical Birth Registers or Pregnancy Register data on pregnancy and birth, the national Cause of Death Registers, and incident reporting. Further details about linkage frequency and data sources are listed in Table 1, noting that only Finland can link the abortion register to the Cause of Death register. National confidential enquiries are based on retrieved information from clinical records, and the national findings have been combined on a Nordic level for publication since 2005.^3^, ^4^, ^5^, ^6^ The NOSS national teams originally collected clinical information about severe adverse maternal morbidity directly from obstetric institutions, targeting specified conditions or interventions. Evolving surveillance models currently combine data from the national Medical Birth Registers or Pregnancy Register, national patient registers, and may also include validation through clinical records which provide additional clinical information in each country. Results are subsequently combined.^7^, ^8^, ^9^, ^10^, ^11^

**TABLE 1:** Data collection for maternal deaths and severe maternal morbidity in the Nordic Maternal Mortality Collaboration and the Nordic Obstetric Surveillance Studies.

	Denmark	Finland	Iceland	Norway	Sweden
**Maternal deaths up to 1 year after end of pregnancy**					
Linkage interval	3 years	1 year	1 year	1 year	1 year
Data sources	Medical Birth Register^a^ Cause of Death Register	Medical Birth Register^a^ Cause of Death Register^b^ Incident reporting	Medical Birth Register^a^ Cause of Death Register Incident reporting	Medical Birth Register^a^ Cause of Death Register Incident reporting	Pregnancy Register Cause of Death Register Incident reporting
Clinical review	All cases	All cases	All cases	All cases	All cases
**Severe maternal morbidity**					
Linkage	Project based	Project based	Project based	Project based	Project based
Data sources	Medical Birth Register Hospital discharge codes	Medical Birth Register Hospital discharge codes	Medical Birth Register Hospital discharge codes	Medical Birth Register (MBRN) Hospital discharge codes	Pregnancy Register, from first visit to hospital discharge.
COVID‐19	Register linkage Incident reporting	Register linkage Incident reporting	Register linkage Incident reporting	Incident reporting to the MBRN	Incident reporting

^a^ The Nordic medical birth registers—a potential goldmine for clinical research—Langhoff‐Roos—2014—Acta Obstetricia et Gynecologica Scandinavica—Wiley Online Library.

^b^ In Finland, it is also possible to link the abortion and cause of death registers for research purposes to identify maternal deaths in early pregnancy (https://thl.fi/en/statistics‐and‐data/statistics‐by‐topic/sexual‐and‐reproductive‐health/parturients‐deliveries‐and‐births/perinatal‐statistics‐parturients‐delivers‐and‐newborns).

Safe governance of health data is essential to protect sensitive information but may also represent barriers to surveillance and learning. Following the publication of the European General Data Protection Regulation (GDPR) in 2016, strict interpretations in several countries prevented international sharing of individual health information. The new regulations also increased the costs of health register data, which was a challenge for the Nordic surveillance during the COVID‐19 pandemic. These restrictions as well as costs were associated with reduced register‐based research in Finland, although new legal amendments allow sharing of (pseudo)anonymized research data internationally if the host country regulations are compatible to EU/EEA requirements. Currently, Nordic maternal morbidity and mortality studies use aggregated national data. With aligned definitions of outcomes and covariates, new methods for meta‐analysis are possible, but permanent data‐sharing regulations and infrastructure for joint surveillance and learning are lacking.

However, Nordic, European, and global initiatives for secondary use of health data have explored the needs related to infrastructure for sharing data and developed common data models and programmed federated analyses where individual health information is not shared across institutions or borders. This contrasts with conventional analyses based on a single, centralized dataset. The Euro‐Peristat collaboration has developed a model for perinatal surveillance using joint R‐scripts and federated analyses of routine data from health registries for key indicators of maternal and perinatal health. Similar solutions for federated data analysis could be adapted for severe maternal morbidity and mortality but require validation of rare and severe adverse outcomes.

New digital interfaces can facilitate data collection, safe processes for learning, and dissemination of learning outcomes. In the INOSS collaboration, the Belgian Obstetric Surveillance Study/Belgian Analysis System for Maternal Mortality has an advanced safe interface for digital data collection, audit, and dissemination of results (BAMM | B‐OSS). Following audit, health leaders and clinicians can access information about learning outcomes related to their role and place of work, but they have no access to the individual data.

Similarly, current research projects in Finland and Norway aim to build national, digital systems for audits of maternal deaths or severe morbidity, also including retrieval of clinical information from medical records. In Norway, pilot studies on eclampsia and severe maternal infections are ongoing.^12^ The clinical data are directly stored at a safe data server and imported into an expert review interface; the expert reviewers will see the anonymized clinical information and can fill in the evaluation form, note learning outcomes and review their own previous assessments. Summary information from all reviews is stored and can be accessed by the administrator. Anonymized clinical information, learning outcomes, and review summaries are stored separately and not in a conventional single file dataset. The latter is important to maintain anonymity and ensure learning as a group outcome‐ in contrast to medicolegal reviews of single cases.

All the Nordic countries and several INOSS countries established population‐based surveillance of pregnant women admitted to hospital with COVID‐19 infection during the pandemic.^13^ Aligned definitions and data collection forms were essential to compare incidence and outcomes across countries. The Nordic studies indicated a protective effect of public health interventions reducing transmission during the first two pandemic waves.^14^ The INOSS studies also identified medical undertreatment of pregnant women who needed respiratory support, and potentially more severe perinatal outcomes in births to women with moderate to severe COVID‐19.^15^ The Nordic collaboration utilized different organizational models for this surveillance, and future preparedness could be improved by “dormant” preapproved emergency protocols and established infrastructure for registry utilization, collection of clinical information and data sharing across the countries. This aligns with a recently published roadmap for research on maternal and perinatal health in the context of epidemic threats published by the World Health Organization (WHO), which calls for research on epidemiology, social vulnerability, access to prevention with vaccines, and medical treatment.

Use of routine data in surveillance and research may reduce clinicians' burden of reporting.

All Nordic countries have well established Medical Birth Registers and have national patient data from secondary specialist care. While the first NOSS studies and maternal mortality studies were based on clinical data collection independent of the registers, this structure required considerable effort from clinicians and resulted in under‐reporting. Recently, studies from the NOSS and the Nordic Maternal Mortality Collaboration have been based on register linkages and subsequent collection of supplementary clinical information from medical records. This enables recording more details than the routine variables reported to the national registers. Verification and validation are essential for rare and severe outcomes based on routine data sources. Automated retrieval of relevant clinical information could reduce the burden of reporting further; the Swedish Pregnancy Register automatically receives updated information from the electronic health records. However, utilization of health register data for surveillance of severe maternal morbidity and mortality is hampered by linkage fees, which are particularly high in Denmark and Finland, and time‐consuming application and linkage processes represent major barriers. Better routine linkages as part of the national surveillance infrastructure could enable rapid and targeted analyses and mitigate missed learning opportunities in the current structure.

In conclusion, ongoing Nordic research collaborations in surveillance of and learning from maternal deaths and severe morbidity have contributed to improved healthcare during the last decades. With small maternity populations in each country, collaboration is essential for robust surveillance of rare, severe outcomes. The Nordic countries have high‐quality health data registers on pregnancy and childbirth and established scientific and clinical collaborations, but we need a joint effort to move from single research projects on severe maternal and perinatal outcomes to collaborative, cross‐national routine surveillance and learning from deaths and severe morbidity, to improve maternal and perinatal health.

## AUTHOR CONTRIBUTIONS

The authors are national PIs and have contributed to the conceptualization and writing of this editorial. All authors have approved the final version.

## FUNDING INFORMATION

HME contributed to this work with funding from the Norwegian Research Council (grant no 320181) and acted as coordinating PI for the Nordic COVID‐19 in pregnancy grant from the Nordic Federation of Societies of Obstetrics and Gynecology (NFOG) fund (grant no 6506). The PIs of the Nordic Maternal Mortality Group and the NOSS group have received support from the NFOG fund for meeting and travel costs.

## CONFLICT OF INTEREST STATEMENT

The authors report no conflict of interest.

## Data Availability

The data that support the findings of this study are available on request from the corresponding author. The data are not publicly available due to privacy or ethical restrictions.
